# Development of dendrite polarity in Drosophila neurons

**DOI:** 10.1186/1749-8104-7-34

**Published:** 2012-10-30

**Authors:** Sarah E Kargbo-Hill, Manpreet Parmar, Kyle W Gheres, Michelle A Guignet, Yanmei Huang, F Rob Jackson, Melissa M Rolls

**Affiliations:** 1 Biochemistry and Molecular Biology, The Pennsylvania State University, 118 Life Sciences, University Park, PA 16802 USA; 2 Tufts Center for Neuroscience Research, Tufts University School of Medicine, Boston, MA 02111 USA

**Keywords:** Branch Point, Fluorescence Recovery After Photobleaching, RNAi Line, Mixed Polarity, Mammalian Neuron

## Abstract

**Background:**

Drosophila neurons have dendrites that contain minus-end-out microtubules. This microtubule arrangement is different from that of cultured mammalian neurons, which have mixed polarity microtubules in dendrites.

**Results:**

To determine whether Drosophila and mammalian dendrites have a common microtubule organization during development, we analyzed microtubule polarity in Drosophila dendritic arborization neuron dendrites at different stages of outgrowth from the cell body *in vivo*. As dendrites initially extended, they contained mixed polarity microtubules, like mammalian neurons developing in culture. Over a period of several days this mixed microtubule array gradually matured to a minus-end-out array. To determine whether features characteristic of dendrites were localized before uniform polarity was attained, we analyzed dendritic markers as dendrites developed. In all cases the markers took on their characteristic distribution while dendrites had mixed polarity. An axonal marker was also quite well excluded from dendrites throughout development, although this was perhaps more efficient in mature neurons. To confirm that dendrite character could be acquired in Drosophila while microtubules were mixed, we genetically disrupted uniform dendritic microtubule organization. Dendritic markers also localized correctly in this case.

**Conclusions:**

We conclude that developing Drosophila dendrites initially have mixed microtubule polarity. Over time they mature to uniform microtubule polarity. Dendrite identity is established before the mature microtubule arrangement is attained, during the period of mixed microtubule polarity.

**Electronic supplementary material:**

The online version of this article (doi:10.1186/1749-8104-7-34) contains supplementary material, which is available to authorized users.

## Background

Many neurons are specialized to receive information through dendrites and send signals through axons. In order to establish and maintain functionally distinct compartments, different proteins must accumulate in axons and dendrites. Most neuronal protein synthesis takes place in the cell body[1], and so proteins must be moved to their place of function after synthesis. This long-distance transport is primarily microtubule-based[2–4], and so the arrangement of microtubules has the potential to control directional trafficking and neuronal polarity.

In primary cultures of mammalian neurons, microtubules in axons and dendrites have different arrangements. In axons, all microtubules have their plus ends directed away from the cell body (plus-end-out), and thus only plus end-directed kinesins can carry cargo into axons[1, 5]. In contrast, dendritic microtubules have mixed polarity[6, 7], and so any motor protein could carry cargo into dendrites.

In addition to the fundamental observation that microtubules have different arrangements in axons and dendrites, there are several studies that indicate a strong link between microtubule organization and dendrite identity. In cultured hippocampal neurons four to five unspecified neurites initially extend from the soma; all of these processes have plus-end-out microtubules[1]. One of the processes then begins a rapid growth phase, and this process becomes the axon. Later, the remaining processes initiate growth as dendrites. The time at which many dendritic features are acquired and dendrites start their major outgrowth coincides with the time at which minus-end-out microtubules are added to the initial population of plus-end-out microtubules[8]. Further evidence for the link between minus-end-out microtubules and dendrite identity was provided by phenotypic analysis of neurons in which levels of MKLP1 were reduced. In these neurons the number of minus-end-out microtubules in dendrites went down after antisense RNA treatment to reduce MKLP1, and at the same time dendritic shape and contents, including ribosomes, were lost[9]. Thus there seems to be a strong link between mixed dendrite microtubule polarity and dendrite identity in cultured mammalian neurons.

However, the dendrites in which microtubules have been studied most extensively *in vivo* do not have mixed polarity. Drosophila neurons have axons and dendrites with polarized structure and function similar to mammalian neurons[10]. Like mammalian neurons, Drosophila neurons have axons with uniform plus-end-out microtubules[11]. However, dendrites of Drosophila larval sensory neurons[11–13], motor neurons[11], and interneurons[11] have close to uniform minus-end-out polarity. Motor neuron dendrites in C. elegans neurons share this minus-end-out organization[14]. This discrepancy in microtubule organization between cultured mammalian neurons and Drosophila and C. elegans neurons raises a number of questions, including whether polarized trafficking in invertebrate and mammalian neurons is fundamentally different, and whether a uniform minus-end-out neurite is capable of outgrowth. To begin to answer these questions we have analyzed the development of microtubule polarity in Drosophila neurons *in vivo*. We find that, as in mammalian neurons, Drosophila dendrites have mixed polarity early in their outgrowth. In fact it takes several days after dendrites are specified to attain uniform minus-end-out polarity. We also find that, as in mammalian neurons, major features of dendrite identity are established when dendritic microtubules are mixed.

## Results

### Strategies for live imaging of neuronal microtubule polarity during embryonic and larval development

**Figure 1:**
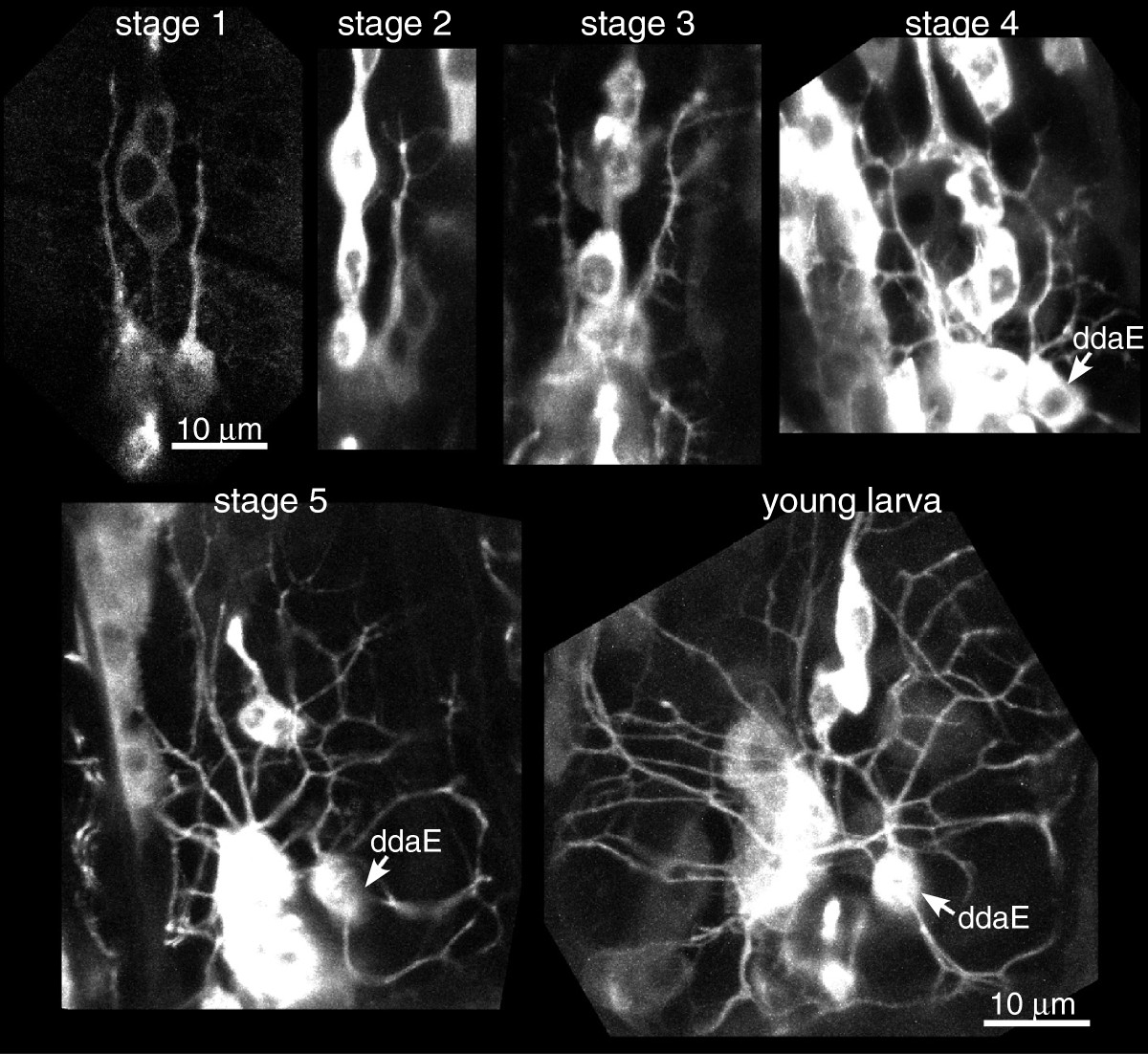
**Morphology of dendritic arborization neurons during dendrite development.** EB1-GFP was expressed in stage 2+ neurons with elav-Gal4 and representative images from different stages during dendrite outgrowth are shown. 1407-Gal4 was used to express EB1-GFP in stage 1 neurons. These stages are used for reference throughout the study. ‘Young larva’ refers to an animal collected within 2 h of hatching. Stage 1 refers to neurons with linear dendrites extending from the cell body; at stage 2 branches are seen to initiate; at stage 3 branches are longer, and secondary branches initiate; at stage 4 more secondary branches are visible. Stage 5 neurons have complex branched dendrites.

To determine how microtubule polarity is established in dendrites of Drosophila neurons, we first needed to develop imaging conditions that would allow us to assay microtubule growth at all stages of dendrite development. Growing, but not shrinking, microtubules can be labeled with end-binding (EB) protein family members tagged with fluorescent proteins. The direction of movement of EB protein comets seems to give a reliable readout of microtubule polarity, and has been used to monitor polarity in mammalian, C. elegans, and Drosophila neurons[5, 7, 11, 14]. In Drosophila, microtubule dynamics are most easily assayed in larval dendritic arborization neurons[11–13, 15]. These are sensory neurons that extend dendrites under the cuticle and epidermal cells and act as proprioceptors and nociceptors[16, 17]. Their cell bodies are located in the periphery at the center of their often elaborate dendritic arbors and their axons extend to the central nervous system. As their dendrites are very close to the surface of the animal, they are well-suited for live imaging. We therefore used these neurons for our analysis. One of the simplest of these neurons, ddaE, is visible from early developmental stages (Figure1), and was used for much of the analysis. In particular, it extends a dendrite that resembles a comb, and localization studies were performed in this dendrite.

In Drosophila, EB1-GFP can be expressed in neurons or subsets of neurons using the Gal4-UAS system. Most Gal4 drivers we tried did not, however, drive sufficient levels of EB1-GFP to detect comets in neurons at early stages of dendrite development. We did, however, find that 1407-Gal4 could be used to express EB1-GFP at early stages of dendrite outgrowth in Drosophila embryos (Figure1, stage 1). For analysis of later stages of dendrite development we used the pan-neuronal elav-Gal4 driver (Figure1).

For imaging microtubule dynamics in early stages of dendrite outgrowth before embryos were motile, we used a very simple mounting and imaging strategy. Dechorionated embryos under a thin layer of halocarbon oil were imaged on open coverslips suspended on the stage of an upright confocal microscope. This technique allowed us to image dendrites of neurons expressing EB1-GFP until late in embryonic development when embryos start to move inside the vitelline membrane. To image late-stage embryos, corresponding to stage 5 of dendrite development, we developed a strategy to immobilize living embryos.

To reduce mobility of late-stage embryos, we crossed a temperature-sensitive paralytic mutation (*Shi*^*ts1*^) into a genetic background that contained elav-Gal4 and UAS-EB1-GFP transgenes. Embryos with two copies of *Shi*^*ts1*^ and one copy of each of the transgenes were mounted for imaging, and were then warmed on the microscope stage with an objective heater. This allowed us to image EB1-GFP dynamics in paralyzed embryos that had developed up until imaging with normal neuronal function.

Imaging young larvae also presented a challenge as they are much more fragile than the second and third instar larvae we previously analyzed. We therefore used the *Shi*^*ts1*^ line for very young larvae as well and mounted these between an air-permeable membrane and cover slip. We were thus able to perform live imaging of microtubule dynamics throughout dendrite development (Figure1).

### Microtubule polarity progresses from mixed to uniform as dendrites mature

**Figure 2:**
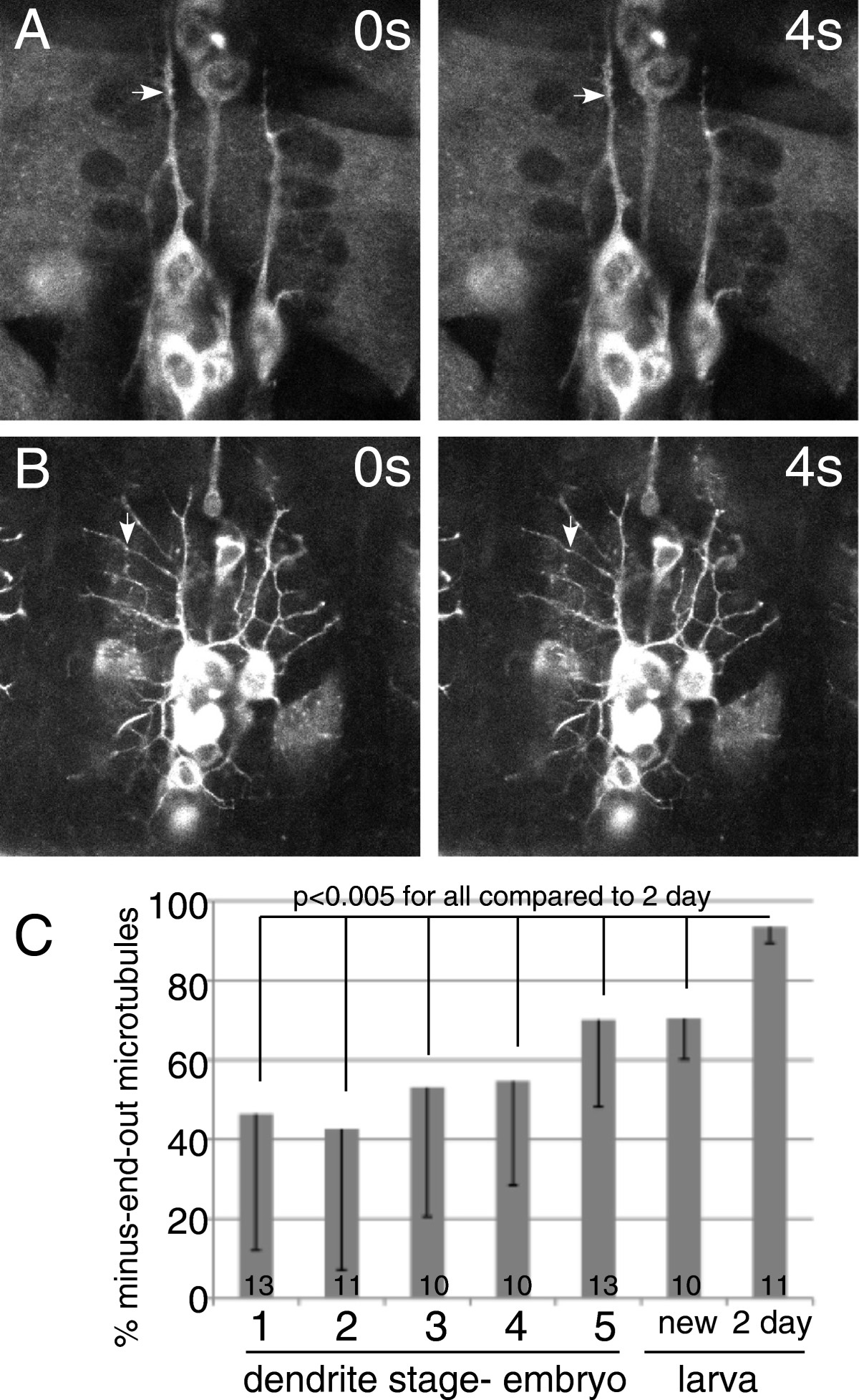
**Microtubule polarity is initially mixed in dendrites and gradually resolves to minus-end-out.** EB1-GFP was expressed in da neurons using elav-Gal4. Movies of EB1-GFP comets were acquired at all stages of dendrite development, and direction of comet movement was analyzed. Individual frames from movies are shown as examples. A stage 1 neuron is shown in (**A**), and stage 5 in (**B**). Arrows point to EB1-GFP comets. Quantitation of comet direction is shown in (**C**). Numbers on bars indicate number of animals analyzed at each time point. The percent of minus-end-out microtubules was calculated from each movie (one movie per animal), and then the averages and standard deviations were calculated at each stage. Standard deviations are shown as the downward error bars. Statistical significance was calculated with an unpaired t-test. Example movies from all stages are shown as Additional files1,2,3,4,5,6,7).

To analyze microtubule polarity throughout dendrite development, we acquired time series of EB1-GFP at the stages indicated in Figures1 and2. At all stages we analyzed neurons in at least 10 different animals. Example frames from these movies are shown (Figure2 and Additional files1,2,3,4,5,6,7). Growing microtubules appear as bright spots that can be tracked through multiple frames of the movie. The direction of comet movement was determined from the movies, and percent of minus-end-out microtubules in each movie were calculated. The averages and standard deviations were calculated from the movies taken for each stage (Figure2C). During early stages of dendrite outgrowth, from stage 1 when dendrites extended from the cell body as a single unbranched process, to the establishment of much more branched dendrites in stage 4, 40% to 50% of microtubules had minus-end-out polarity. At these time points there were some dendrites in which comets moved primarily in one direction, and this could be either plus-end-out or minus-end-out, and there were also many dendrites which were mixed, with comets moving in opposite directions (see Additional files2 and3). Generally variability and mixing characterized these early stages. At late embryonic and early larval stages the percentage of minus-end-out microtubules increased to about 70%. Again, individual dendrites with comets moving in both directions were observed (see Additional file5). The mature almost uniform and very consistent minus-end-out polarity was attained by 2 days after larval hatching. Thus the transition from mixed polarity to uniform minus-end-out microtubules in dendrites was gradual and was not complete until well after dendrites were highly branched and the animal had been living as a functional larva.

### Ribosomes localize to dendrites while microtubules are mixed

The late stage in dendrite development at which uniform polarity was acquired suggested that dendrite identity was established while microtubule polarity was mixed. Ribosomes localize predominantly to the cell body and dendrites in mammalian neurons, and very few are present in axons under normal circumstances[1]. As ribosomes can therefore be used as markers of dendrites, we wished to determine whether ribosomes localized to Drosophila dendrites before or after they acquired uniform microtubule polarity.

**Figure 3:**
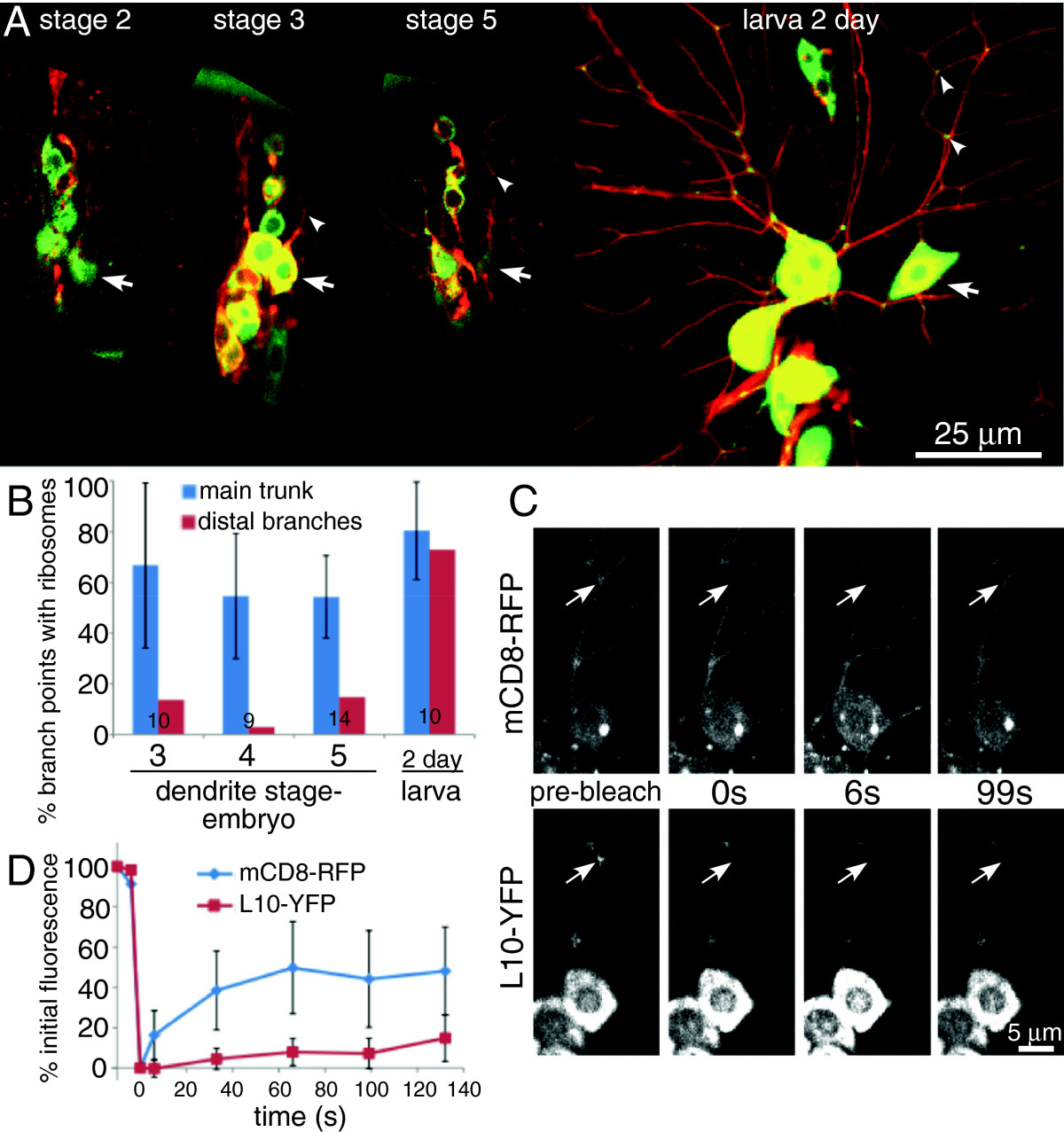
**Ribosomal protein L10 localizes to dendrite branch points throughout dendrite development.** The ribosomal protein L10 tagged with YFP was expressed in neurons together with mCD8-RFP to mark cell shape. Patterns of fluorescence were analyzed at different stages of dendrite development. Examples from different stages are shown in (**A**). Arrows point out the cell body of the ddaE neuron, and arrowheads point to spots of YFP at dendrite branch points. (**B**) The percent of proximal branch points occupied by ribosomes was counted. The percent of branch points occupied was calculated for each cell. The average of these numbers is shown in the graph. The error bars show the standard deviation. The data on occupancy of distal branch points were pooled from all cells as numbers were smaller. Numbers on the bars indicate the number of animals analyzed at each time point. (**C**) An example of a FRAP experiment is shown. The same ddaE cell is shown in the top and bottom rows. An arrow points to the area that was bleached. In the pre-bleach image a bright spot of L10-YFP is present at the branch point that was bleached. Fluorescence of both RFP and YFP is strongly reduced after bleaching. However, by 6 s the mCD8-RFP has started to return to the bleached area. Very little L10-YFP returned to the bleach area during the time course. (**D**) The average fluorescence recovery of nine cells is shown in the graph. Error bars show standard deviations.

To label ribosomes, we expressed the ribosomal subunit L10 tagged with YFP in neurons. This tagged protein was previously shown to have a similar localization pattern to other components of the protein synthetic machinery in Drosophila brains, highly concentrated in the cell body and absent from axons[15]. In da neurons most L10-YFP was seen in cell bodies (Figure3A). We also observed distinct spots of L10-YFP at dendrite branch points (Figure3A). To determine whether these were likely to represent dendritic ribosomes, we performed fluorescence recovery after photobleaching (FRAP) experiments. If L10-YFP was not incorporated into ribosomes, it would be expected to diffuse very rapidly and recover quickly after photobleaching. If the tagged protein was attached to a ribosome it would be expected not to recover much as these are large structures that diffuse little in neurons[18]. When spots of L10-YFP at dendrite branch points were bleached, very little recovery was observed in 2 min after bleaching (Figure3C and D). In contrast the membrane marker mCD8-RFP, which was bleached at the same time, recovered rapidly (Figure3C and D). L10-YFP therefore is likely to represent ribosome distribution. To gain further support for the validity of L10-YFP as a marker of ribosomal localization, we compared its distribution to that of an independently generated tagged ribosomal subunit. L10a is a ribosomal protein that has no sequence similarity to L10. Mouse L10a, which is about 80% identical to the fly L10a protein, was tagged with EGFP and expressed in da neurons. Like L10-YFP, EGFP-L10A was localized to the cytoplasm in the cell body and many dendrite branch points (average branch point occupancy 61%, standard deviation 17.2% in 17 larvae examined; example images are shown in Additional file8: Figure S1). Bright spots were also seen in many nuclei; these are probably nucleoli. Thus both ribosomal markers examined localized to dendrite branch points, and so we determined when in development they took up this position.

When we examined the pattern of L10-YFP fluorescence during dendrite development, we could observe spots at branch points as soon as they were formed (Figure3A and B). Initially L10-YFP was primarily at branch points along main trunks of dendrites that emerged from cell bodies. Two days after larval hatching spots of fluorescence were also seen at more distal branch points (Figure3 A and B). This increase in distal L10-YFP could either reflect increased concentrations of L10-YFP that make all ribosome populations easier to visualize, or it could reflect an outward transport of ribosomes as uniform polarity in dendrites is achieved. In either case, L10-YFP was capable of localizing to major dendrite branch points at the time when microtubule polarity was mixed. We thus conclude that ribosome targeting to dendrites occurs during mixed microtubule polarity, and does not require uniform minus-end-out polarity.

### Mitochondria and the dendritic protein Apc2 can localize to dendrite branch points before microtubules attain uniform polarity

**Figure 4:**
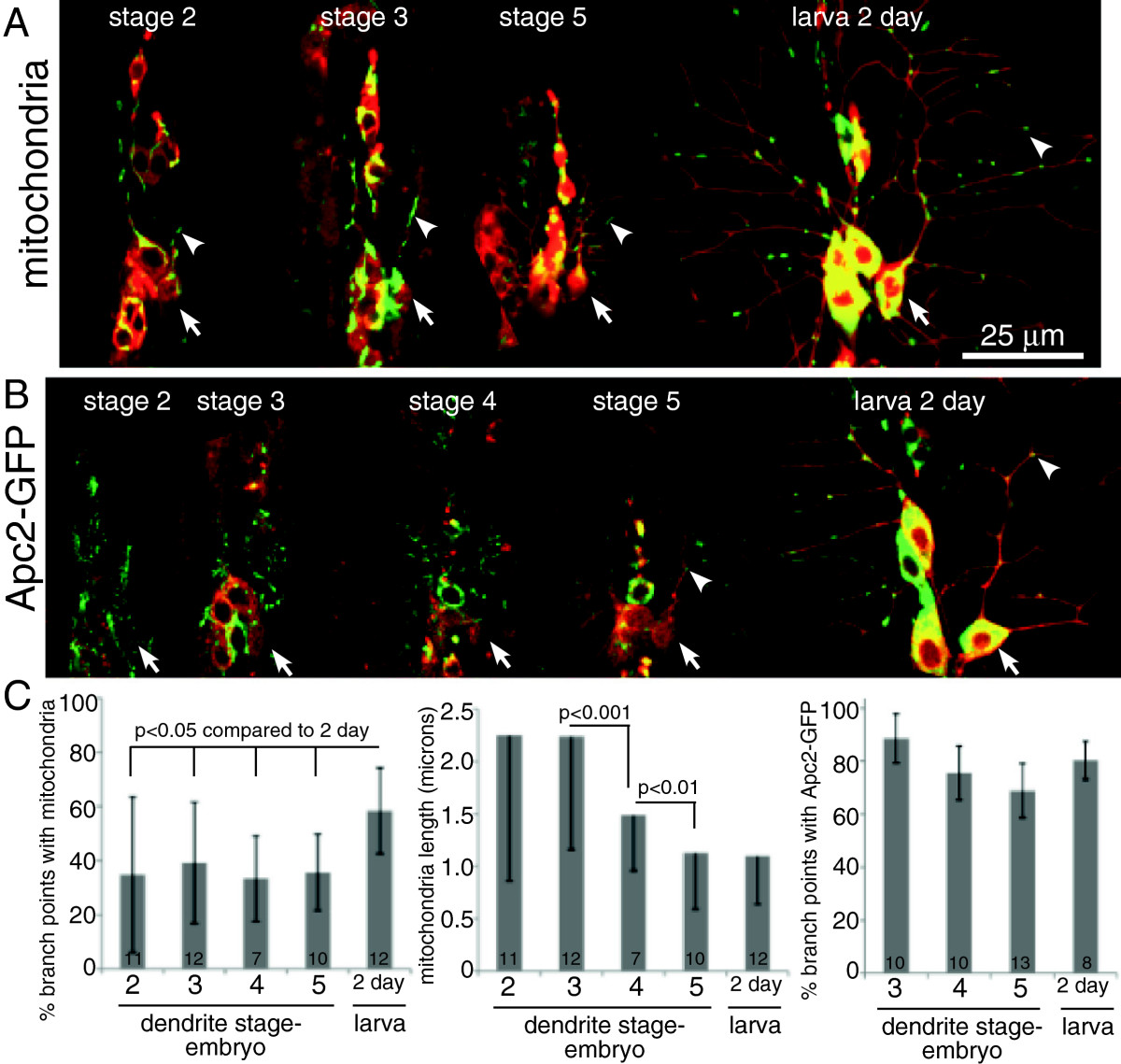
**Mitochondria and Apc2-GFP have characteristic localizations in dendrites throughout development.** (**A**) Mitochondria were labeled with mitoGFP and cell shape was outlined with mCD8-RFP. Images of mitochondrial localization in living embryos and larvae were acquired at different stages of development. (**B**) Apc2-GFP was expressed in neurons together with mCD8-RFP. Images of dendrites at different stages were acquired in whole, living embryos or larvae. (**C**) Images of mitochondria or Apc2-GFP were used to quantitate the percent of ddaE branch points occupied with green fluorescence. Only branch points along the main trunk of the comb dendrite of ddaE were counted. % occupancy was calculated for each cell, and averages are shown. The error bars show standard deviations. Mitochondria lengths were also measured at different stages of neuronal development (middle graph); averages are shown with standard deviations indicated by the error bars. Numbers on the bars indicate numbers of animals analyzed at each time point. Statistical significance was calculated with a t-test.

To confirm that uniform microtubule polarity was not a prerequisite for localization of organelles or proteins to specific regions of dendrites, we tracked mitochondria and Apc2-GFP (adenomatous polyposis coli 2) during dendrite development. Mitochondria are distributed throughout axons and dendrites[19], and in dendrites often seem to be associated with branch points (Figure4A and C). While the proportion of branch points occupied by mitochondria increases slightly 2 days after larval hatching, around 40% of branch points are occupied from the time they are formed (Figure4A and C). The most obvious change in mitochondria during dendrite development was a change in shape. When dendrites initially extended from the cell body most mitochondria had an extended linear shape. As development proceeded, mitochondria become shorter (Figure4A and C). This change, however, is more likely to be related to altered mitochondrial fission than organization of microtubules. Mitochondria took on their specific position within dendrites early in development, but we also wanted to test another marker that was polarized to dendrites and not found in axons.

Apc2-GFP is localized to cell bodies, dendrites, and some proximal axons, but not distal axons, in Drosophila central neurons[15] and dendritic arborization neurons[20]. It is one of the most polarized markers we have found that distinguishes Drosophila dendrites from axons. Within dendrites it is very specifically localized to dendrite branch points where Apc2 plays a role in controlling microtubule polarity[21]. Apc2-GFP localized strongly to dendrites from very early stages (Figure4B and C). It also localized to nascent branches as they were forming, from the stage when new branches resemble filopodia. As the branches developed, Apc2-GFP became more distinctly localized to the branch point itself. Thus localization of Apc2-GFP in dendrites, and association with branches, was a very early developmental event that preceded uniform microtubule polarity.

### Mitochondria and Apc2-GFP distribute normally in dendrites with mixed microtubule polarity

As ribosomes, mitochondria and Apc2-GFP took their characteristic positions in dendrites before uniform microtubule polarity was established, we hypothesized that mixed microtubule polarity was sufficient to determine dendrite identity. To test whether uniform microtubule polarity was required for localization of organelles or proteins within dendrites, we compared marker distribution in control neurons and neurons with reduced levels of Kap3. Kap3 is an accessory subunit of the kinesin-2 motor[22, 23]. When levels of Kap3 or either of the two motor subunits of kinesin-2 are reduced, dendrite microtubule polarity is non-uniform[21]. In the main trunk of the class I dendritic arborization neuron ddaE microtubule polarity changes from less than 5% plus-end-out to greater than 30% plus-end-out when Kap3 is targeted by RNAi[21]. Dendrite shape is unchanged under these conditions[21]. We therefore compared mitochondria and Apc2-GFP distribution in control and Kap3 RNAi ddaE neurons.

**Figure 5:**
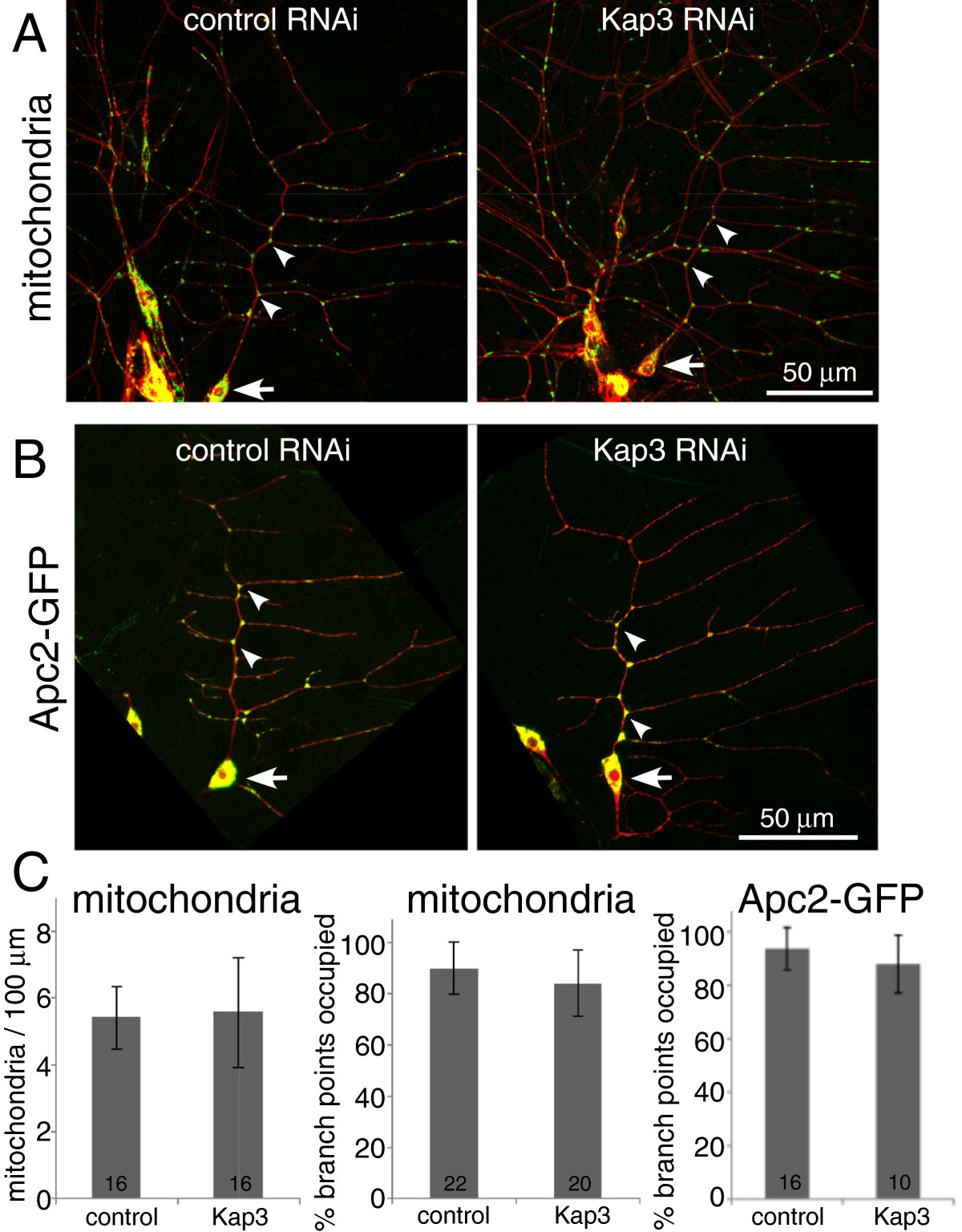
**Mitochondria and Apc2-GFP localize to dendrite branch points in larvae with mixed dendritic microtubules.** (**A**) Control (rtnl2) or Kap3 RNA hairpins were expressed in neurons together with mCD8-RFP, dicer2, and mitoGFP. Images of neurons in living larvae were acquired and mitochondria distribution in dendrites was analyzed. Arrows point to the cell body of ddaE and arrowheads to mitochondria at branch points along the comb dendrite. (**B**) Control (rtnl2) or Kap3 RNA hairpins were expressed with Apc2-GFP, dicer2, and mCD8-GFP in class I da neurons with 221-Gal4. Example images are shown; arrows point to ddaE cell bodies and arrowheads to punctae of Apc2-GFP at branch points along the main trunk of the ddaE comb dendrite. (**C**) In the left panel, the number of mitochondria per 100 μm of dendrite length is shown. The length of the main trunk of the ddaE neuron was measured, and the number of mitochondria in this part of the dendrite arbor was counted. A value was generated for each cell analyzed, and the average of these is shown in the graph. Error bars are standard deviations of these averages. In the middle panel the percentage of branch points along the comb dendrite of ddaE occupied by mitochondria is shown. Again, percentages were generated for each neuron and the averages and standard deviations were graphed. The right panel shows a similar analysis of branch points occupied by Apc2-GFP. In all graphs the number of animals analyzed for each genotype is shown on the bar.

The overall distribution of mitochondria in control RNAi and Kap3 RNAi neurons appeared similar (Figure5A). To quantitatively compare the mitochondrial pattern in the comb dendrite of ddaE in these two genotypes we used two measures. We counted the number of mitochondria per unit length of dendrite, and found that in control and Kap3 RNAi dendrites about five mitochondria were present per 100 microns of dendrite (Figure5B). We also counted the percent of branches along the main comb dendrite of ddaE that were occupied by mitochondria. In both genotypes 80% to 90% of these branch points contained mitochondria (Figure5). These numbers are likely higher than those for developing dendrites (Figure4C) for two reasons: (1) only branch points along the main dendrite trunk of ddaE were counted here, compared to all visible branch points in developing dendrites; and (2) the images in Figure5 were acquired later in development than the last data point in Figure4.

Similar experiments were performed to determine whether Apc2-GFP localized differently in mature neurons with uniform or mixed polarity (Figure5B). In control RNAi neurons more than 90% of branch points along the main trunk of the ddaE comb dendrite contained Apc2-GFP punctae. Similar numbers of branch points were occupied when Kap3 levels were reduced by RNAi (Figure5C). Thus two different markers were able to occupy their normal positions in dendrites that cannot acquire uniform microtubule polarity. This result is consistent with the experiments in embryos that showed Apc2-GFP and mitochondria localized to dendrites early in their development, before uniform microtubule polarity was established.

### Few ANF-GFP vesicles are found in dendrites, even when microtubules have mixed polarity

**Figure 6:**
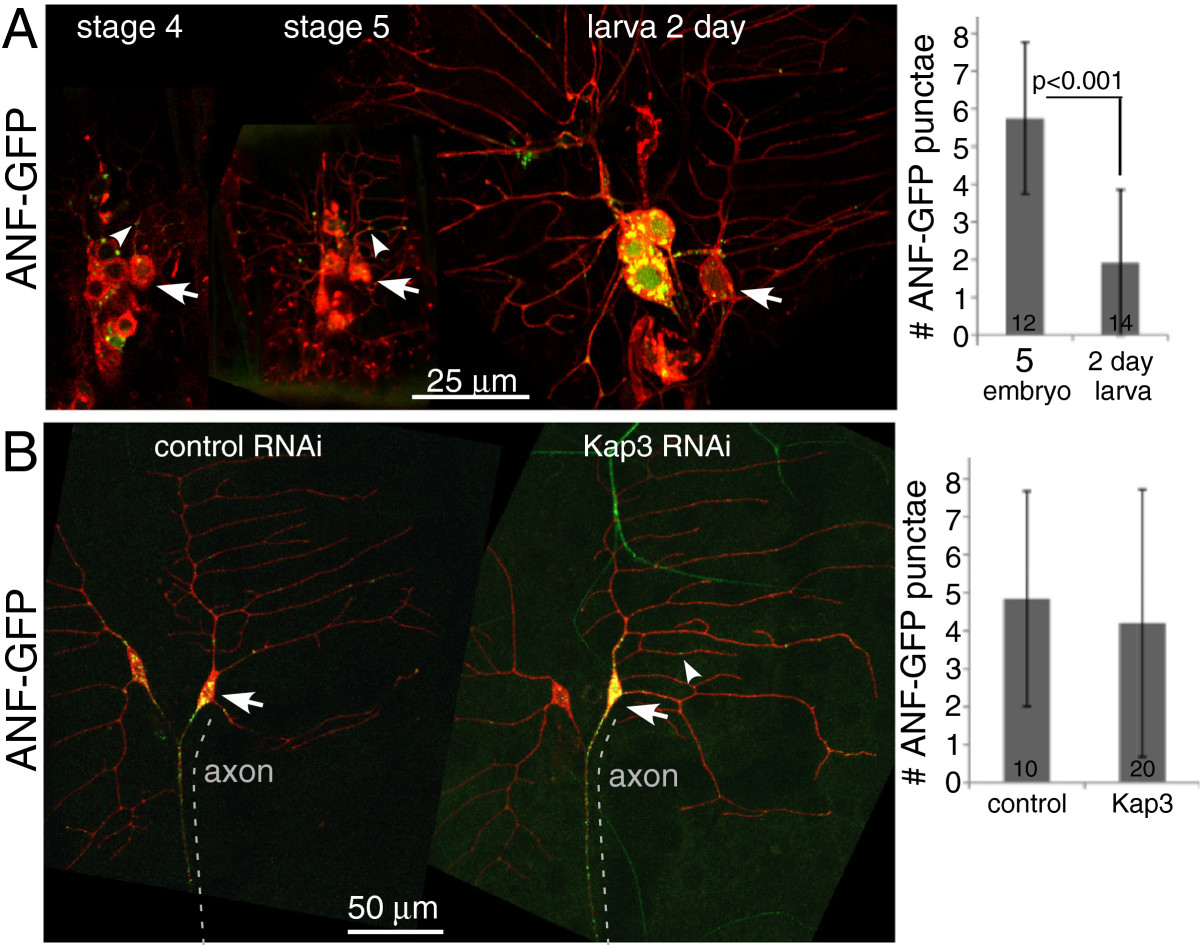
**Few ANF-GFP punctae are localized to dendrites.** (**A**) To localize ANF-GFP at different stages of dendrite development, it was expressed in all neurons together with mCD8-RFP using elav-Gal4. Examples of embryos at stage 4 and 5 are shown, as well as 2-day-old larvae. The ddaE neuron cell body is indicated with an arrow and examples of ANF-GFP punctae are indicated with arrowheads. To compare the numbers of ANF-GFP punctae in embryonic and larval dendrites, the total number of punctae in the dorsal comb-shaped dendrite of the ddaE neuron was counted in stage 5 neurons and neurons of 2-day-old larvae. The average number of punctae was calculated. The error bars show the standard deviation, the number of cells analyzed is listed within the bars on the graph, and the *P* value was calculated with a t-test. (**B**) ANF-GFP was expressed in class I da neurons with the 221-Gal4 driver in conjunction with mCD8-RFP, dicer2, and large hairpin RNAs targeting either Rtnl2 (control) or Kap3. After overnight collection, animals were aged for 3 days at 25°C before imaging. Example images of both genotypes are shown. The number of ANF-GFP punctae in the ddaE comb dendrite beyond the first branch point was counted. Averages and standard deviations are shown in the graph.

As dendritic components localized to dendrites early in development and did not seem to require uniform polarity microtubules to establish their characteristic distributions, we also wished to determine whether axonal components could be excluded from axons while dendritic microtubules were mixed. EGFP-tagged Atrial Natriurietic Factor (ANF-GFP) has been used to perform detailed tracking of dense core vesicles (DCVs) in Drosophila neurons[24]. When this marker is expressed in dendritic arborization neurons punctae are abundant in the cell body and axons of larvae, but are rare in dendrites (Figure6B). To determine when this polarized distribution is established we analyzed ANF-GFP localization in embryonic da neurons. Individual punctae only became bright enough to score consistently in neurons with branched dendrites (stage 3–4). At this time punctae were visible in the cell body, and could also be seen in some dendrites (Figure6A). In stage 5 neurons, most ddaE neurons had at least three punctae of ANF-GFP in the comb dendrites (Figure6A). Although the dendrite arbors of 2-day larvae were much longer than those in embryos, the number of punctae did not increase, and in fact was slightly lower (Figure6A). We therefore considered the possibility that the establishment of uniform microtubule polarity might help to exclude axonal cargoes from dendrites.

To test the link between uniform microtubule polarity and exclusion of DCVs from dendrites, we tested whether the number of ANF-GFP punctae would increase in neurons in which Kap3 levels were reduced and dendrite microtubule polarity was mixed. In both Kap3 and control RNAi neurons in 3-day-old larvae, ANF-GFP punctae were frequently found in dendrites up to the first branch point. However, only a few punctae were typically found beyond this point in either genotype (Figure6B), and no significant difference between genotypes was observed. Note that the number of punctae in larvae in Figure6B is higher than in 6A, probably because a stronger Gal4 driver was used in the RNAi experiment. We conclude that DCVs may be more slightly efficiently excluded from dendrites in mature neurons, but this does not seem to be due to the more uniform microtubule polarity.

## Discussion

This study is the first to examine how microtubule polarity develops in dendrites *in vivo*. In Drosophila dendritic arborization neurons, microtubule polarity was mixed during the major period of dendrite outgrowth during embryogenesis. Towards the end of embryogenesis and the beginning of larval life, the number of minus-end-out microtubules gradually exceeded that of plus-end-out microtubules, until 2 days into the larval period more than 90% of microtubules were minus-end-out. In mammalian cultured neurons, microtubules also have mixed polarity in dendrites as they grow out from the cell body[8]. Thus microtubule polarity is similar in fly and mammalian dendrites during their development. This similarity suggests that conserved mechanisms could establish the basic layout of neuronal microtubules from flies to mammals.

To determine whether the change from mixed to uniform polarity in Drosophila dendrites affects localization of organelles or proteins, we analyzed a variety of markers during dendrite development. All were able to acquire their characteristic localization patterns in developing dendrites with mixed polarity. Again, this suggests similarity with mammalian neurons in culture, which can develop polarized dendrites with mixed microtubule orientation[1]. Moreover specific cargoes can be delivered directly to mixed orientation dendrites in culture by polarized transport[25]. Thus mixed microtubule polarity is sufficient to allow targeting of dendritic cargoes in both systems.

For analysis of axonal targeting we used ANF-GFP as it labels discrete neuropeptide vesicles that can be targeted relatively specifically to axons in da neurons (see Figure6B). During embryonic dendrite development some of these vesicles could be seen in dendrite arbors, and when the marker was expressed at relatively low levels with elav-Gal4 (Figure6A) very few were seen in larval dendrites. Thus DCVs may be slightly more efficiently excluded from dendrites in more mature neurons. To probe whether efficient exclusion of DCVs from dendrites might be related to acquisition of uniform microtubule polarity we compared the number of punctae in dendrites with mixed and uniform polarity microtubules. In both cases occasional punctae were seen in dendrites, but there was not a difference between the two sets. The lack of DCV escape into dendrites with mixed polarity is consistent with a recent study of DCV targeting to C. elegans dendrites, which also found that factors other than microtubule polarity were likely to control polarized targeting of these vesicles[14]. It is possible, however, that microtubule polarity may contribute to efficient exclusion of other types of axonal cargoes from dendrites.

Two major models of polarized transport have been proposed to account for differential accumulation of proteins and organelles in axons and dendrites. One relies on plus end-directed kinesins to transport cargo into both axons and dendrites. In this model either different sets of motors, or different motor-cargo combinations are directed specifically to axons or dendrites[2, 3]. This type of kinesin-based model could account for trafficking in developing dendrites of flies and mammals, but becomes difficult to rationalize in mature Drosophila dendrites. The alternate model is that the major minus end-directed motor, dynein, plays a key role in transport from the cell body into dendrites. Indeed there is phenotypic evidence for a role for dynein in anterograde dendrite transport in Drosophila[12, 13], and dynein can also transport cargo into mammalian dendrites[26]. It will be interesting to determine the relative contributions of kinesins and dynein to dendrite trafficking in future studies.

The finding that dendrite identity is established in flies and mammals when microtubules have mixed polarity raises the question of why Drosophila dendrites switch to minus-end-out polarity. The only current answers to this question are speculative. Having opposite polarity microtubules in axons and dendrites should increase efficiency of polarized transport and reduce targeting errors. So far we have not been able to detect differences in Drosophila dendrites with mixed and uniform polarity, but these may exist.

It is not yet clear whether mammalian dendrites ever have uniform minus-end-out polarity. One intriguing study has raised the possibility that very mature mammalian neurons *in vivo* may have regions of dendrites near the cell body that have uniform microtubule polarity. However, the second harmonic generation microscopy technique used could not distinguish between uniform polarity plus-end-out or minus-end-out microtubules[27], so it remains to be resolved whether mammalian dendrites can have minus-end-out polarity. It is clear, however, that developing Drosophila and mammalian neurons share a similar organization of microtubules, and that in both cases roughly equal numbers of plus and minus-end-out microtubules are present as dendrites grow.

## Conclusions

We have analyzed microtubule polarity and marker distribution during development of dendrites in Drosophila neurons in vivo. We conclude that as they grow out from the cell body, dendrites initially have mixed microtubule polarity. This organization gradually changes to minus-end-out over the next two days. The dendritic and axonal markers examined were able to localize correctly before the final microtubule polarity was achieved.

## Methods

### Fly stocks and genetics

Flies were kept at room temperature in standard media. Most transgenic lines have been previously described (see[15]). Many of the lines were received from the Bloomington Drosophila Stock Center. RNAi lines were from the Vienna Drosophila RNAi Center (VDRC). A UAS-ANF-GFP insertion on the third chromosome was generously provided by David Deitcher.

The only line not previously described was the ribosomal marker UAS-EGFP-L10a. As a basis for this line, a plasmid containing an inframe fusion of EGFP and the mouse L10a ribosomal protein was obtained from Joshua Ainsley (Tufts University School of Medicine). The EGFP-L10a coding segment was subcloned into the pUAST transformation vector, and transgenic strains were generated using standard methods by Genetic Services, Inc.

In order to visualize microtubule polarity several different genetic backgrounds were used. For early stages of dendrite outgrowth 1407 Gal4 was used to drive expression of UAS-EB1-GFP. For older embryos and larvae, progeny from a cross between males of genotype shi^ts1^/Y; elav- Gal4, UAS-EB1-GFP and females of genotype shi^ts1^/shi^ts1^ were used. In order to visualize ribosomes in the da neurons, males with UAS-mcD8-RFP on chromosome II were crossed with females of the line UAS-L10-YFP, elavGal4/TM6 and non-tubby progeny were imaged. To visualize mitochondria, larvae from the line UAS-dicer, UAS-mcD8-RFP/CyO; elav-Gal4, UAS-mitoGFP/TM6 were used. Lastly, to visualize Apc2-GFP, males with an elav-Gal4 transgene were crossed to females from the line UAS-mCD8-RFP, UAS-dicer2; 221-Gal4, UAS-Apc2-GFP. This Apc2-GFP line was also used as a tester line for RNAi experiments, and progeny from this line crossed to a control RNAi targeting Rtnl2 (VDRC 33320) or an experimental RNAi targeting Kap3 (VDRC 45400) were analyzed. For analysis of mitochondria localization in conjunction with RNAi the UAS-dicer, UAS-mcD8-RFP/CyO; elav-Gal4, UAS-mitoGFP/TM6 line was crossed to the same pair of RNAi lines and progeny were analyzed. For analysis of ANF-GFP localization a tester line of genotype UAS-dicer, UAS-mcD8-RFP/CyO; 221-Gal4, UAS-ANF-GFP/TM6 was generated. It was crossed to RNAi lines targeting either Rtnl2 as a control or Kap3. For RNAi analysis embryos were collected overnight on either a yeasted apple cap or a cap containing standard Drosophila media, then aged for 3 days at 25°C.

### Embryo and larval preparation and microscopy

Embryos were collected overnight on apple caps with yeast paste. The chorion was dissolved with 50% bleach solution for 2 min. After thorough rinsing with water, embryos were collected in heptane and transferred to a coverslip. Immediately after the heptane evaporated, embryos were covered in a thin layer of halocarbon oil 27 (Sigma). The coverslip was suspended with embryos open underneath it on the microscope stage. For EB1-GFP imaging in late stage embryos, an objective heater (BiOptechs) set to 36°C was used to warm the embryos as they were being imaged.

Young larvae were collected from apple caps within 2 h after hatching. They were then placed on air-permeable membrane supported on a metal slide and a coverslip was placed on top. An objective heater was used as for embryos.

For ‘2 day’ larvae imaging, embryos were collected overnight on a food cap. The food cap was then removed to a petri dish and larvae were aged for two days at 25°C before imaging. Larvae were then rinsed in Schneider’s media and transferred onto dried agarose pad on microscope slides. Larvae were then carefully rotated to dorsal side up and covered with glass coverslips taped onto the slide.

An Olympus FV1000 confocal microscope was used for all imaging. Timeseries were acquired using a 60× 1.4 NA objective.

### Analysis of EB1-GFP, mitoGFP, L10-YFP, and Apc2-GFP

For each embryo or larva, neurons in only one hemisegment were analyzed. All analysis was performed in ImageJ. For EB1-GFP dynamics, comets were only counted if seen in three consecutive frames. Each dendrite branch was watched independently for comets. Dendrites were categorized as those emerging directly from the cell body and those branching from another dendrite. For mito-GFP and Apc2-GFP, spots on branch points were counted as well as total number of branch points in the dendrites. These values were used to determine percentage of branch points occupied. For mito-GFP spot length was also recorded for every spot visible in the dendrites. The ImageJ measure function was used to determine length.

### FRAP analysis

Photobleaching of L10-YFP was performed with the 488 nm laser on an Olympus FV1000 confocal microscope. Both L10-YFP and mCD8-RFP were bleached simultaneously with this laser at 100% power. Images of both channels were acquired every 3.3 s after bleaching. Analysis of recovery was performed in ImageJ. Small regions of interest within the bleach area, and also a background area without a fluorescent cell, were defined and the average intensity of the area was measured in the time series. The background value was subtracted from the value in the bleach area. The value of the area of interest was set to 100 before bleaching and 0 immediately after bleaching. Remaining values were normalized to this scale. Averages of these normalized values are plotted in the graph. Error bars represent the standard deviation at each timepoint.

### RNAi and analysis of Apc2-GFP and mitoGFP

Progeny of the analysis lines for Apc2-GFP and mitoGFP (see section on fly stocks and genetics) crossed to RNA hairpin lines were collected overnight on food caps. Caps were then transferred to a petri dish and aged for 3 days at 25°C. Larvae were mounted for live imaging on slides with dried agarose pads, and coverslips were taped on top of the larvae to restrain them. Images were acquired with an Olympus FV1000 confocal microscope. The localization of Apc2-GFP and mitoGFP was analyzed in the ddaE neuron. One ddaE neuron per animal was imaged and analyzed. Branch point occupancy was calculated along the primary branch of the dorsal comb-like dendrite. Mitochondria per unit length was also calculated in this part of the neuron. Measurements were performed using ImageJ.

### Analysis of ANF-GFP

For developmental analysis ANF-GFP was expressed together with mCD8-RFP using the pan-neuronal elav-Gal4 driver. Images were acquired as for other markers. For RNAi analysis the tester line UAS-mCD8-RFP, UAS-dicer2; UAS-ANF-GFP, 221-Gal4 was crossed to either Rtnl2 (VDRC 33320) or Kap3 (VDRC 45400) RNAi lines. Larvae were aged as for other RNAi experiments, and GFP punctae beyond the first branch point of the ddaE comb dendrite were counted in images acquired on a Zeiss LSM510 microscope.

## Electronic supplementary material

Additional file 1: Movie 1. EB1-GFP in embryos: stage 1. EB1-GFP was expressed in embryos with 1407-Gal4. Movies were acquired with a confocal microscope. Cell bodies (CB) are near the bottom of the frame, and the dendrites extend dorsally. Examples of comets are labeled with stars in the movie. (MOV 5 MB)

Additional file 2: Movie 2. EB1-GFP in embryos: stage 2. EB1-GFP comets can be seen in dendrites extending from the cell bodies in this cell that is late stage 1/stage 2. Several examples are labeled with stars. Cell bodies (CB) are at bottom left. (MOV 7 MB)

Additional file 3: Movie 3. EB1-GFP in embryos: stage 3. EB1-GFP was expressed in neurons with elav-Gal4. An example of neurons as they are extending side branches from dendrites is shown. Several comets are marked with stars. (MOV 6 MB)

Additional file 4: Movie 4. EB1-GFP in embryos: stage 4. EB1-GFP was expressed with elav-Gal4. Several examples of comets are indicated with stars. Many of the bright cells down the middle of the group of cells are neurons with ciliated dendrites, not branched dendrites as in the dendritic arborization neurons we analyzed. (MOV 8 MB)

Additional file 5: Movie 5. EB1-GFP in embryos: stage 5. EB1-GFP was expressed in all neurons with elav-Gal4. Again, central bright cells are ciliated sensory neurons that do not extend branched dendrites. Examples of comets in dendritic arborization neuron dendrites are indicated with stars. Ripples in the movie are from muscle contractions; although the animals had a temperature sensitive paralytic allele, they were not completely immobilized under the imaging conditions. (MOV 6 MB)

Additional file 6: Movie 6. EB1-GFP in a newly hatched larva. EB1-GFP expressed by elav-Gal4 was imaged in animals less than 2 h after hatching. The overall shape of the neurons is very similar to late stage embryos. Several comets are labeled with stars. (MOV 7 MB)

Additional file 7: Movie 7. EB1-GFP in a larva 2 days after hatching. Dendrites from a single neuron expressing EB1-GFP are visible in this movie. The cell body is at top right, so comets moving up are at the tips of minus-end-out microtubules. Examples of comets are labeled with stars. (MOV 2 MB)

Additional file 8: Figure S1. EGFP-L10a localizes to the cell body and dendrite branch points of da neurons. EGFP-L10a and mCD8-RFP were expressed in all neurons with elav-Gal4. Images of da neurons 2-day-old larvae were acquired and two examples are shown. EGFP-L10a concentrations localized at dendrite branch points are indicated with arrowheads. (PDF 2 MB)

## Authors’ original submitted files for images

Below are the links to the authors’ original submitted files for images. Authors’ original file for figure 1 Authors’ original file for figure 2 Authors’ original file for figure 3 Authors’ original file for figure 4 Authors’ original file for figure 5 Authors’ original file for figure 6

## Abbreviations

Apc: Adenomatous polyposis coli
EB: End-binding
FRAP: Fluorescence recovery after photobleaching.
