# Supplementary figures and images for: Development of dendrite polarity in Drosophila neurons
Source: Neural Dev. 2012 Oct 30;7:34. doi: 10.1186/1749-8104-7-34 (PMC3570434; doi:10.1186/1749-8104-7-34)

ribosomes (L10a)

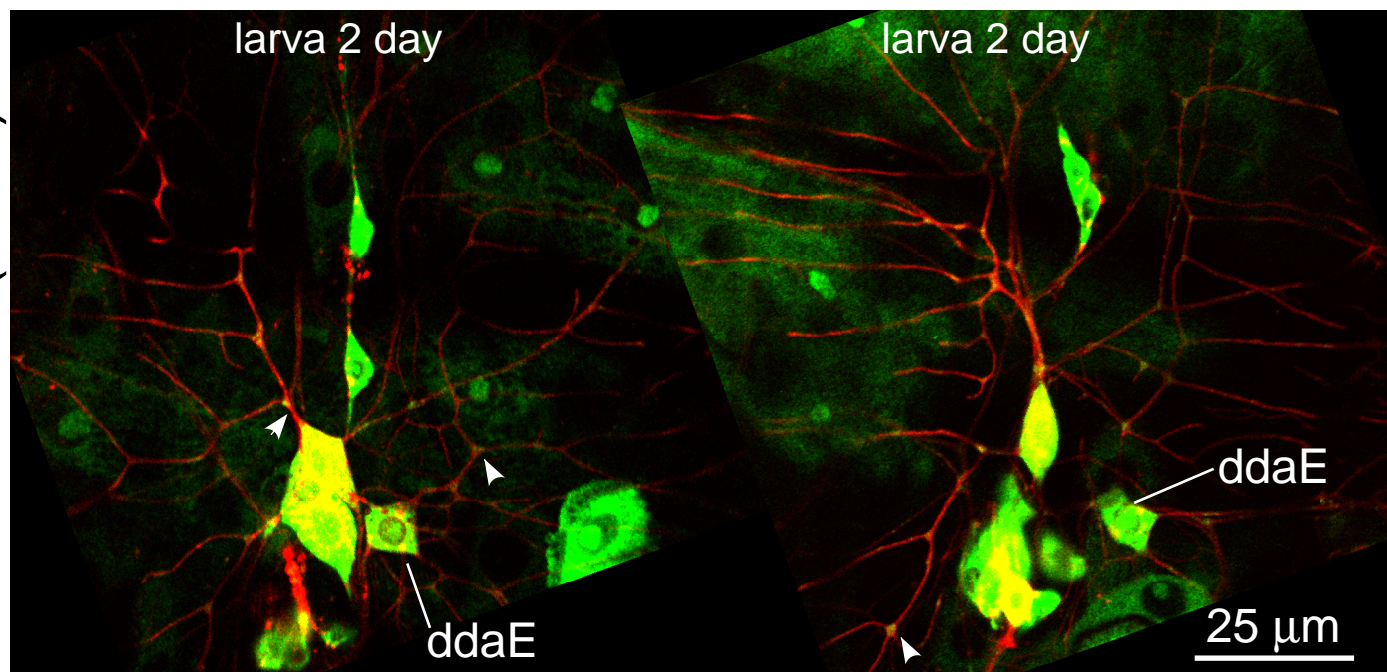

Supplement: Supplementary file 8 — Additional file 8: Figure S1. EGFP-L10a localizes to the cell body and dendrite branch points of da neurons. EGFP-L10a and mCD8-RFP were expressed in all neurons with elav-Gal4. Images of da neurons 2-day-old larvae were acquired and two examples are shown. EGFP-L10a concentrations localized at dendrite branch points are indicated with arrowheads. (PDF 2 MB) [file 13064_2012_223_MOESM8_ESM.pdf]

stage 1

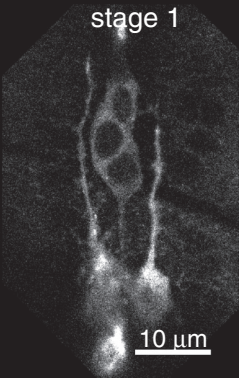

stage 2

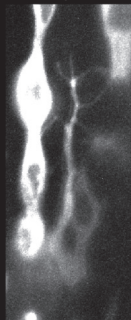

stage 3

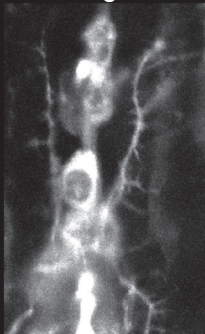

stage 4

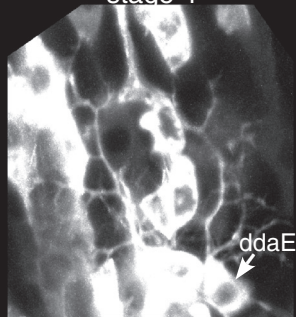

stage 5

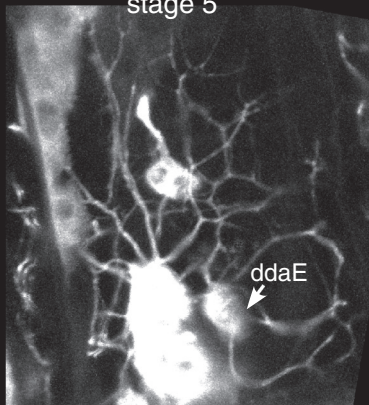

young larva

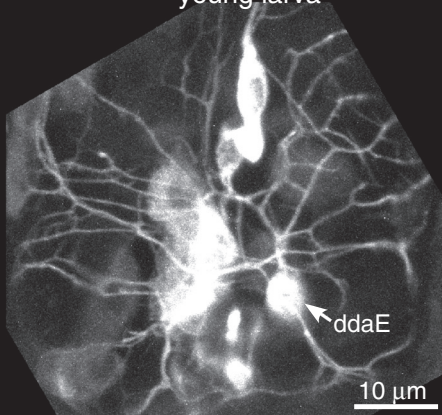

Supplement: Supplementary file 9 — Authors’ original file for figure 1 [file 13064_2012_223_MOESM9_ESM.pdf]

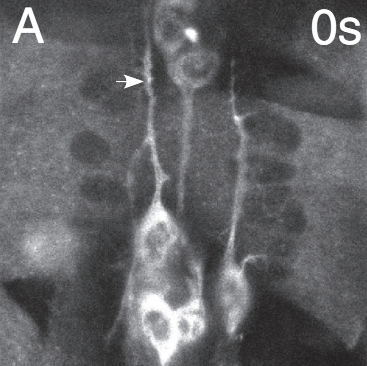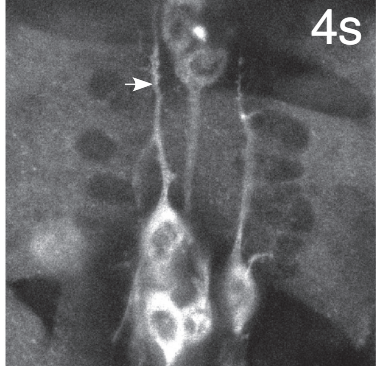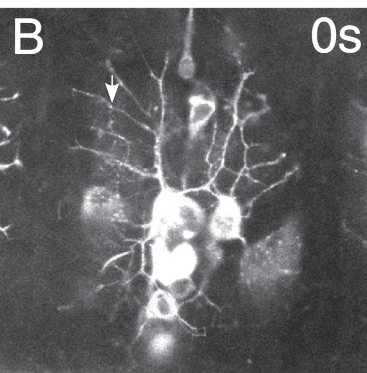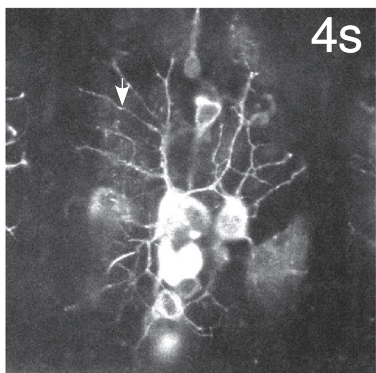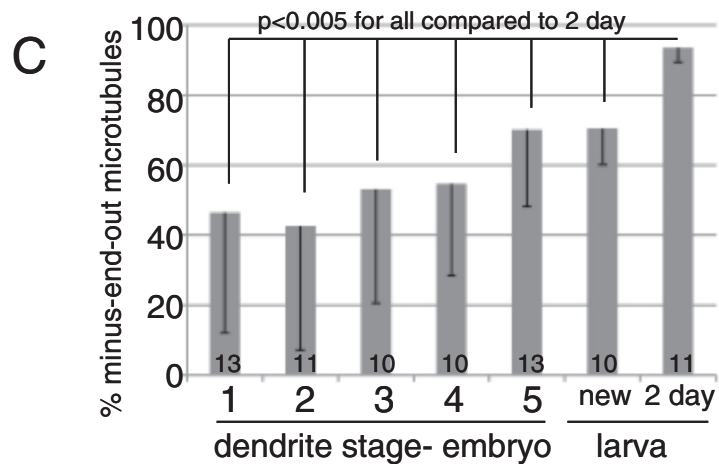

Supplement: Supplementary file 10 — Authors’ original file for figure 2 [file 13064_2012_223_MOESM10_ESM.pdf]

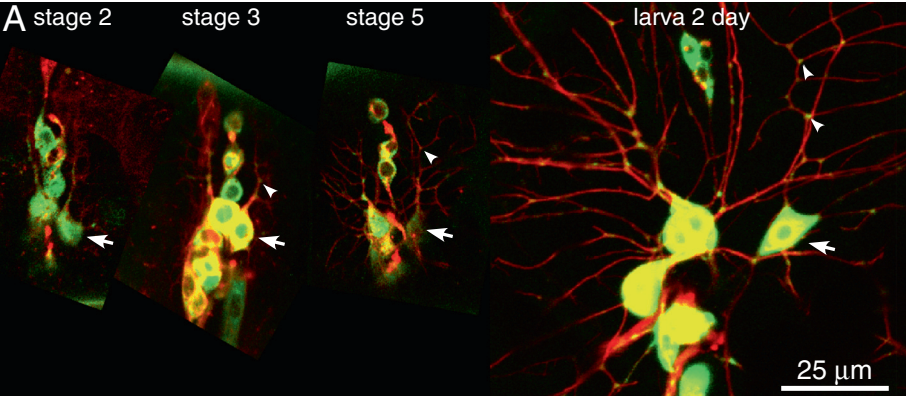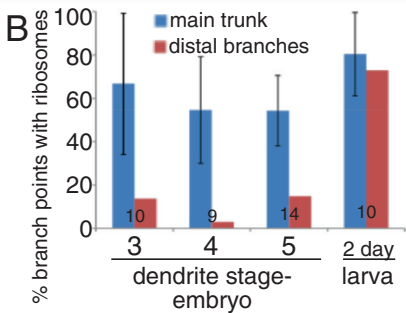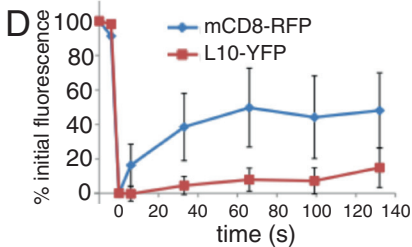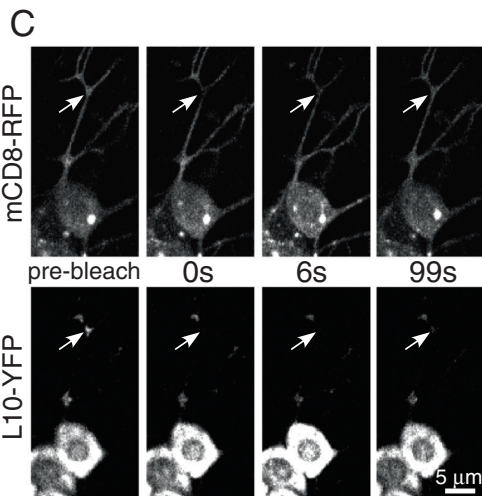

Supplement: Supplementary file 11 — Authors’ original file for figure 3 [file 13064_2012_223_MOESM11_ESM.pdf]

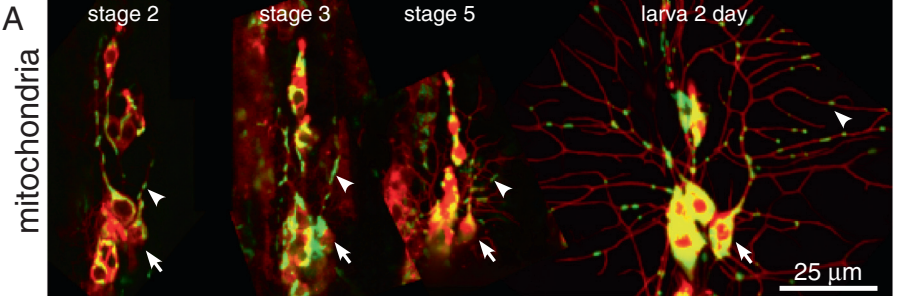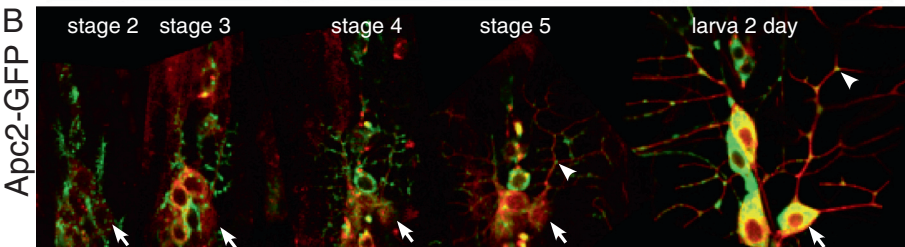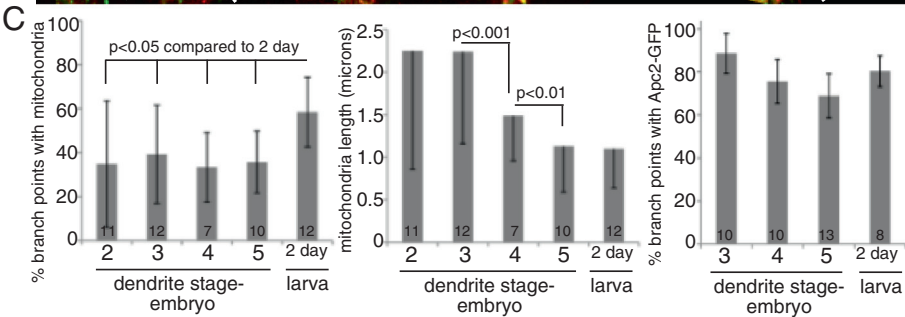

Supplement: Supplementary file 12 — Authors’ original file for figure 4 [file 13064_2012_223_MOESM12_ESM.pdf]

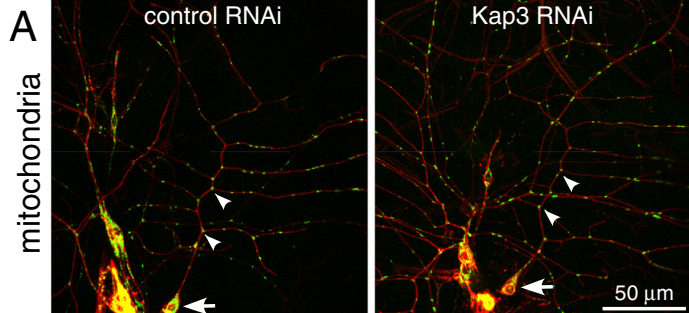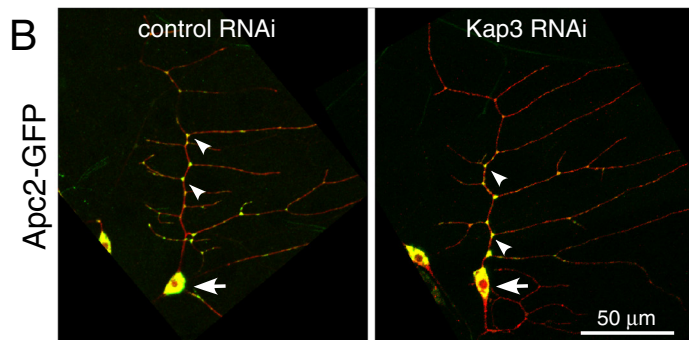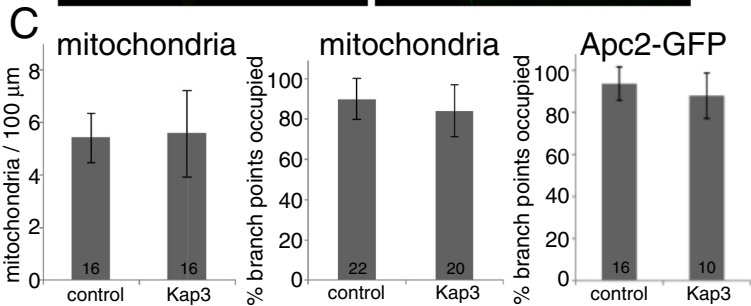

Supplement: Supplementary file 13 — Authors’ original file for figure 5 [file 13064_2012_223_MOESM13_ESM.pdf]

**A**

ANF-GFP

stage 4

stage 5

larva 2 day

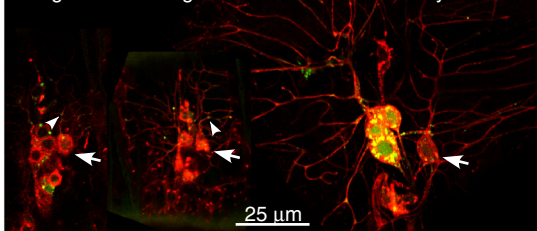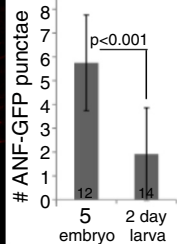**B**

ANF-GFP

control RNAi

Kap3 RNAi

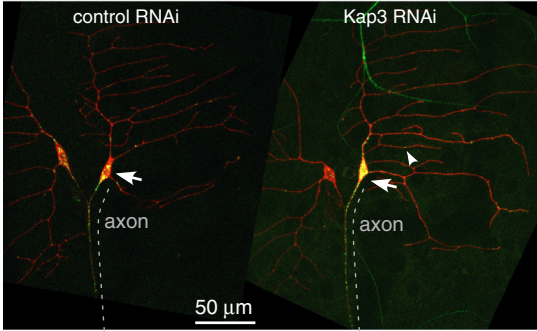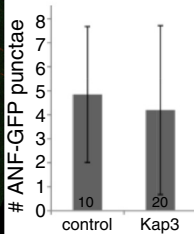

Supplement: Supplementary file 14 — Authors’ original file for figure 6 [file 13064_2012_223_MOESM14_ESM.pdf]
